# 

**Published:** 1915-04

**Authors:** 


PUBLISHED MONTHLY. ATLANTA SUBSCRIPTION $1.00

### JOURNAL-RECORD OF MEDICINE

Successor to { Atlanta Medical and Surgical Journal, Established 1855. and Southern Medical Record, Established 1870. } 62nd Year

Vol. LXII ATLANTA, GA., APRIL, 1915. No. 1

